# The Macroecology of Sustainability

**DOI:** 10.1371/journal.pbio.1001345

**Published:** 2012-06-19

**Authors:** Joseph R. Burger, Craig D. Allen, James H. Brown, William R. Burnside, Ana D. Davidson, Trevor S. Fristoe, Marcus J. Hamilton, Norman Mercado-Silva, Jeffrey C. Nekola, Jordan G. Okie, Wenyun Zuo

**Affiliations:** 1 Department of Biology, University of New Mexico, Albuquerque, New Mexico, United States of America; 2 United States Geological Survey, Fort Collins Science Center, Jemez Mountains Field Station, Los Alamos, New Mexico, United States of America; 3 Santa Fe Institute, Santa Fe, New Mexico, United States of America; 4 Instituto de Ecología, Universidad Nacional Autónoma de México, México Distrito Federal, México; 5 Department of Anthropology, University of New Mexico, Albuquerque, New Mexico, United States of America; 6 School of Natural Resources and the Environment, University of Arizona, Tucson, Arizona, United States of America; Imperial College London, United Kingdom

## Abstract

The discipline of sustainability science has emerged in response to concerns of natural and social scientists, policymakers, and lay people about whether the Earth can continue to support human population growth and economic prosperity. Yet, sustainability science has developed largely independently from and with little reference to key ecological principles that govern life on Earth. A macroecological perspective highlights three principles that should be integral to sustainability science: 1) physical conservation laws govern the flows of energy and materials between human systems and the environment, 2) smaller systems are connected by these flows to larger systems in which they are embedded, and 3) global constraints ultimately limit flows at smaller scales. Over the past few decades, decreasing per capita rates of consumption of petroleum, phosphate, agricultural land, fresh water, fish, and wood indicate that the growing human population has surpassed the capacity of the Earth to supply enough of these essential resources to sustain even the current population and level of socioeconomic development.

## Abstract 2

Global consumption rates of vital resources suggest that we have surpassed the capacity of the Earth to sustain current levels, much less future trajectories of growth in human population and economy.

“Sustainability” has become a key concern of scientists, politicians, and lay people—and for good reason. There is increasing evidence that we have approached, or perhaps even surpassed, the capacity of the planet to support continued human population growth and socioeconomic development [1]–[3]. Currently, humans are appropriating 20%–40% of the Earth's terrestrial primary production [4]–[6], depleting finite supplies of fossil fuels and minerals, and overharvesting “renewable” natural resources such as fresh water and marine fisheries [7]–[10]. In the process, we are producing greenhouse gases and other wastes faster than the environment can assimilate them, altering global climate and landscapes, and drastically reducing biodiversity [2]. Concern about whether current trajectories of human demography and socioeconomic activity can continue in the face of such environmental impacts has led to calls for “sustainability.” A seminal event was the Brundtland commission report [11], which defined “sustainable development (as) development that meets the needs of the present without compromising the ability of future generations to meet their own needs.”

One result has been the emergence of the discipline of *sustainability science*. “Sustainability science (is) an emerging field of research dealing with the interactions between natural and social systems, and with how those interactions affect the challenge of sustainability: meeting the needs of present and future generations while substantially reducing poverty and conserving the planet's life support systems” (*Proceedings of the National Academy of Sciences of the USA* [PNAS], http://www.pnas.org/site/misc/sustainability.shtml). It is the subject of numerous books, at least three journals (*Sustainability Science* [Springer]; *Sustainability*: *Science*, *Practice, & Policy* [ProQuest-CSA]; *International Journal of Sustainability Science and Studies* [Polo Publishing]), and a special section of the PNAS. In “A Survey of University-Based Sustainability Science Programs”, conducted in 2007, (http://sustainabilityscience.org/content.html?contentid=1484), the American Association for the Advancement of Science listed 103 academic programs, including 64 in the United States and Canada, and many more have been established subsequently.

Interestingly, despite the above definition, the majority of sustainability science appears to emphasize social science while largely neglecting natural science. A survey of the published literature from 1980 through November 2010 using the Web of Science reveals striking results. Of the 23,535 published papers that include “sustainability” in the title, abstract, or key words, 48% include “development” or “economics”. In contrast, only 17% include any mention of “ecology” or “ecological”, 12% “energy”, 2% “limits”, and fewer than 1% “thermodynamic” or “steady state”. Any assessment of sustainability is necessarily incomplete without incorporating these concepts from the natural sciences.

## Human Macroecology

A macroecological approach to sustainability aims to understand how humans are integrated into and constrained by the Earth's systems [12]. In just the last 50,000 years, *Homo sapiens* has expanded out of Africa to become the most dominant species the Earth has ever experienced. Near-exponential population growth, global colonization, and socioeconomic development have been fueled by extracting resources from the environment and transforming them into people, goods, and services. Hunter-gatherers had subsistence economies based on harvesting local biological resources for food and fiber and on burning wood and dung to supplement energy from human metabolism. With the transition to agricultural societies after the last ice age [13] and then to industrial societies within the last two centuries, per capita energy use has increased from approximately 120 watts of human biological metabolism to over 10,000 watts, mostly from fossil fuels [3],[14]. Modern economies rely on global networks of extraction, trade, and communication to rapidly distribute vast quantities of energy, materials, and information.

The capacity of the environment to support the requirements of contemporary human societies is not just a matter of political and economic concern. It is also a central aspect of ecology— the study of the interactions between organisms, including humans, and their environments. These relationships always involve exchanges of energy, matter, or information. The scientific principles that govern the flows and transformations of these commodities are fundamental to ecology and directly relevant to sustainability and to the maintenance of ecosystem services, especially in times of energy scarcity [15]. A macroecological perspective highlights three principles that should be combined with perspectives from the social sciences to achieve an integrated science of sustainability.

## Principle 1: Thermodynamics and the Zero-Sum Game

The laws of thermodynamics and conservation of energy, mass, and chemical stoichiometry are universal and without exception. These principles are fundamental to biology and ecology [16]–[18]. They also apply equally to humans and their activities at all spatial and temporal scales. The laws of thermodynamics mean that continual flows and transformations of energy are required to maintain highly organized, far-from-equilibrium states of complex systems, including human societies. For example, increased rates of energy use are required to fuel economic growth and development, raising formidable challenges in a time of growing energy scarcity and insecurity [3],[15],[19]. Conservation of mass and stoichiometry means that the planetary quantities of chemical elements are effectively finite [15],[18].

**Figure 1:**
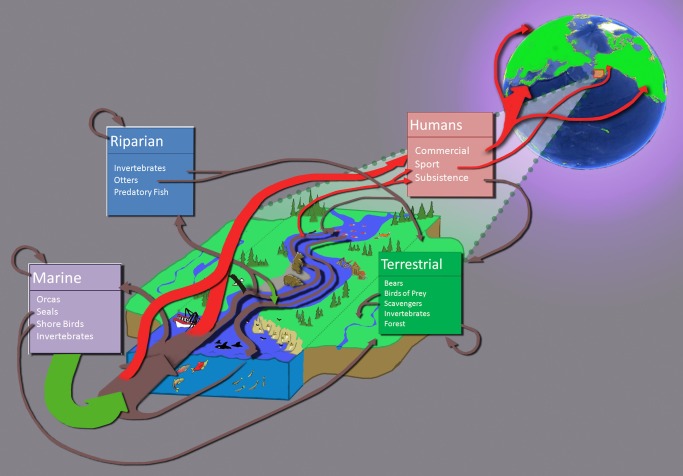
Pictorial illustration of important flows of salmon and contained biomass, energy, and nutrients within and out of the Bristol Bay ecosystem. Brown arrows depict the flows within the ecosystem, green arrows depict inputs due to growth in fresh water or the sea, and red arrows represent human harvest. Seventy percent of salmon are extracted by humans and are no longer available to the Bristol Bay ecosystem.

Human use of material resources, such as nitrogen and phosphorus, alters flows and affects the distribution and local concentrations in the environment [18]. This is illustrated by the Bristol Bay salmon fishery, which is frequently cited as a success story in sustainable fisheries management [20],[21]. In three years for which good data are available (2007–2009), about 70% of the annual wild salmon run was harvested commercially, with one species, sockeye, accounting for about 95% of the catch [22]. From a management perspective, the Bristol Bay sockeye fishery has been sustainable, because annual runs have not declined. Additional implications for sustainability, however, come from considering the effect of human harvest on the flows of energy and materials in the upstream ecosystem (Figure 1). When humans take about 70% of Bristol Bay sockeye runs as commercial catch, this means a 70% reduction in the number of mature salmon returning to their native waters to spawn and complete their life cycles. It also means a concomitant reduction in the supply of salmon to support populations of predators, such as grizzly bears, bald eagles, and indigenous people, all of which historically relied on salmon for a large proportion of their diet [23],[24]. Additionally, a 70% harvest means annual removal of more than 83,000 metric tonnes of salmon biomass, consisting of approximately 12,000, 2,500, and 330 tonnes of carbon, nitrogen, and phosphorus, respectively (see Text S1 for sources and calculations). These marine-derived materials are no longer deposited inland in the Bristol Bay watershed, where they once provided important nutrient subsidies to stream, lake, riparian, and terrestrial ecosystems [24]–[27]. So, for example, one apparent consequence is that net primary production in one oligotrophic lake in the Bristol Bay watershed has decreased “to about 1/3 of its level before commercial fishing” [28]. Seventy percent of Bristol Bay salmon biomass and nutrients are now exported to eastern Asia, western Europe, and the continental US, which are the primary markets for commercially harvested wild Alaskan salmon. Our macroecological assessment of the Bristol Bay fishery suggests that “sustainable harvest” of the focal salmon species does not consider the indirect impacts of human take on critical resource flows in the ecosystem (Figure 1). So the Bristol Bay salmon fishery is probably not entirely sustainable even at the “local” scale.

## Principle 2: Scale and Embeddedness

Most published examples of sustainability focus on maintaining or improving environmental conditions or quality of life in a localized human system, such as a farm, village, city, industry, or country ([29],[30] and articles following [31]). These socioeconomic systems are not closed or isolated, but instead are open, interconnected, and embedded in larger environmental systems. Human economies extract energy and material resources from the environment and transform them into goods and services. In the process, they create waste products that are released back into the environment. The laws of conservation and thermodynamics mean that the embedded human systems are absolutely dependent on these flows: population growth and economic development require increased rates of consumption of energy and materials and increased production of wastes. The degree of dependence is a function of the size of the economy and its level of socioeconomic development [3]. Most organic farms import fuel, tools, machinery, social services, and even fertilizer, and export their products to markets. A small village in a developing country harvests food, water, and fuel from the surrounding landscape.

Large, complex human systems, such as corporations, cities, and countries, are even more dependent on exchanges with the broader environment and consequently pose formidable challenges for sustainability. Modern cities and nation states are embedded in the global economy, and supported by trade and communication networks that transport people, other organisms, energy, materials, and information. High densities of people and concentrations of socioeconomic activities require massive inputs of energy and materials and produce proportionately large amounts of wastes. Claims that such systems are “sustainable” usually only mean that they are comparatively “green”—that they aim to minimize environmental impacts while offering their inhabitants happy, healthy lifestyles.

**Figure 2:**
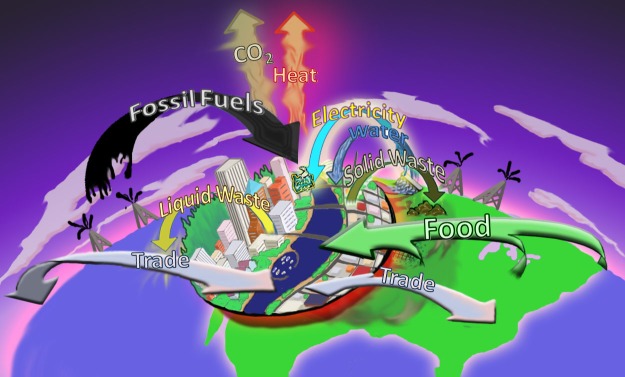
Pictorial illustration of important flows of resources into and wastes out of Portland, Oregon. This “most sustainable city in America” depends on exchanges with the local, regional, and global environments and economies in which it is embedded.

A macroecological perspective on the sustainability of local systems emphasizes their interrelations with the larger systems in which they are embedded, rather than viewing these systems in isolation. Portland, Oregon offers an illuminating example. The city of Portland and surrounding Multnomah County, with a population of 715,000 and a median per capita income of US$51,000, bills itself and is often hailed by the media as “the most sustainable city in America” (e.g., SustainLane.com, 2008). On the one hand, there can be little question that Portland is relatively green and offers its citizens a pleasant, healthy lifestyle, with exemplary bike paths, parks, gardens, farmers' markets, and recycling programs. About 8% of its electricity comes from renewable non-hydroelectric sources (http://apps3.eere.energy.gov/greenpower/resources/tables/topten.shtml). On the other hand, there also can be no question that Portland is embedded in and completely dependent on environments and economies at regional, national, and global scales (Figure 2). A compilation and quantitative analysis of the flows into and out of the city are informative (see Text S1 for sources and calculations). Each year the Portland metropolitan area consumes at least 1.25 billion liters of gasoline, 28.8 billion megajoules of natural gas, 31.1 billion megajoules of electricity, 136 billion liters of water, and 0.5 million tonnes of food, and the city releases 8.5 million tonnes of carbon as CO_2_, 99 billion liters of liquid sewage, and 1 million tonnes of solid waste into the environment. Total domestic and international trade amounts to 24 million tonnes of materials annually. With respect to these flows, Portland is not conspicuously “green”; the above figures are about average for a US city of comparable size (e.g., [32]).

A good way to see the embedding problem is to imagine the consequences of cutting off all flows in and out, as military sieges of European castles and cities attempted to do in the Middle Ages. From this point of view and in the short term of days to months, some farms and ranches would be reasonably sustainable, but the residents of a large city or an apartment building would rapidly succumb to thirst, starvation, or disease. Viewed from this perspective, even though Portland may be the greenest and by some definitions “the most sustainable city in America”, it is definitely not self-sustaining. Massive flows of energy and materials across the city's boundaries are required just to keep its residents alive, let alone provide them with the lifestyles to which they have become accustomed. Any complete ecological assessment of the sustainability of a local system should consider its connectedness with and dependence on the larger systems in which it is embedded.

## Principle 3: Global Constraints

For thousands of years, humans have harvested fish, other animals, and plants with varying degrees of “sustainability” and lived in settlements that depend on imports and exports of energy and materials. Throughout history, humans have relied on the environment for goods and services and used trade to compensate for imbalances between extraction, production, and consumption at local to regional scales. What is different now are the enormous magnitudes and global scales of the fluxes of energy and materials into and out of human systems. Every year fisheries export thousands of tonnes of salmon biomass and the contained energy and nutrients from the Bristol Bay ecosystem to consumers in Asia, Europe, and the US. Every year Portland imports ever larger quantities of energy and materials to support its lifestyle and economy. Collectively, such activities, replicated thousands of times across the globe, are transforming the biosphere.

Can the Earth support even current levels of human resource use and waste production, let alone provide for projected population growth and economic development? From our perspective, this should be ***the*** critical issue for sustainability science. The emphasis on local and regional scales—as seen in the majority of the sustainability literature and the above two examples—is largely irrelevant if the human demand for essential energy and materials exceeds the capacity of the Earth to supply these resources and if the release of wastes exceeds the capacity of the biosphere to absorb or detoxify these substances.

Human-caused climate change is an obvious and timely case in point. Carbon dioxide has always been a waste product of human metabolism—not only the biological metabolism that consumes oxygen and produces carbon dioxide as it converts food into usable energy for biological activities, but also the extra-biological metabolism that also produces CO_2_ as it burns biofuels and fossil fuels to power the maintenance and development of hunter-gatherer, agricultural, and industrial-technological societies. Only in the last century or so, however, has the increasing production of CO_2_ by humans overwhelmed the Earth's capacity to absorb it, increasing atmospheric concentrations and warming the planet more each decade. So, for example, efforts to achieve a “sustainable” local economy for a coastal fishing village in a developing country will be overwhelmed if, in only a few decades, a rising sea level caused by global climate change inundates the community. This shows the importance of analyzing sustainability on a global as well as a local and regional scale.

A macroecological approach to sustainability science emphasizes how human socioeconomic systems at any scale depend on the flows of essential energy and material resources at the scale of the biosphere as a whole. The finite Earth system imposes absolute limits on the ecological processes and human activities embedded within it. The impossibility of continued exponential growth of population and resource use in a finite world has long been recognized [33]–[35]. But repeated failures to reach the limits in the predicted time frames have caused much of the economic establishment and general public to discredit or at least discount Malthusian dynamics. Now, however, there is increasing evidence that humans are pushing if not exceeding global limits [2],[3],[36],[37]. For example, the Global Footprint Network estimates that the ecological footprint, the amount of land required to maintain the human population at a steady state [9], had exceeded the available land area by more than 50% by 2007, and the imbalance is increasing (http://www.footprintnetwork.org/en/index.php/GFN).

**Figure 3:**
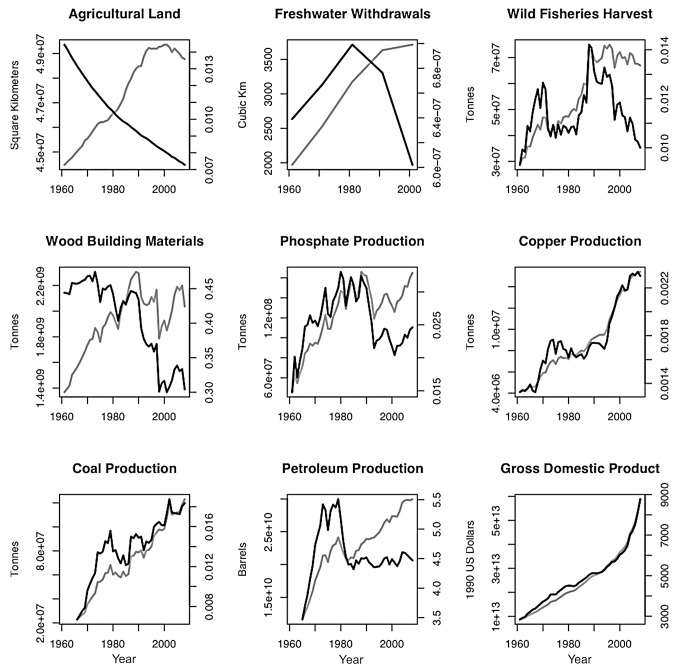
Global trends in total and per capita consumption of resources and GDP from 1961 to 2008. Total global use/production is represented by the grey line using the axis scale on the left side of each diagram. Per capita use/production is represented by the black line using the axis scale on the right side of each diagram. Per capita values represent the total values divided by global population size as reported by the World Resources Institute (http://earthtrends.wri.org/). The *y*-axes are untransformed and scaled to allow for maximum dispersion of variance. Individual sources for global use/production values are as follows: Agricultural land in square-km is from the World Development Indicators Database of the World Bank (http://data.worldbank.org/data-catalog/world-development-indicators) and represents the sum of arable, permanent crop, and permanent pasture lands (see also [46]). Freshwater withdrawal in cubic-km from 1960, 1970, 1980, and 1990 is from UNESCO (http://webworld.unesco.org/water/ihp/db/shiklomanov/part%273/HTML/Tb_14.html) and for 2000 from The Pacific Institute (http://www.worldwater.org/data.html). Wild fisheries harvest in tonnes is from the FAO Fishery Statistical Collection Global Capture Production Database (http://www.fao.org/fishery/statistics/global-capture-production/en) and is limited to diadromous and marine species. Wood building material production in tonnes is based on the FAO ForeSTAT database (http://faostat.fao.org/site/626/default.aspx), and represents the sum of compressed fiberboard, pulpwood+particles (conifer and non-conifer [C & NC]), chips and particles, hardboard, insulating board, medium density fiberboard, other industrial roundwood (C & NC), particle board, plywood, sawlogs+veneer logs (C & NC), sawnwood (C & NC), veneer sheets, and wood residues. Phosphate, copper, and combustible coal production in tonnes is based on World Production values reported in the USGS Historical Statistics for Mineral and Material Commodities (http://minerals.usgs.gov/ds/2005/140/). Global coal production data is limited to 1966–2008. Petroleum production in barrels from 1965 to 2008 is based on The Statistical Review of World Energy (http://www.bp.com/sectiongenericarticle800.do?categoryId=9037130&contentId=7068669) and represents all crude oil, shale oil, and oil sands plus the liquid content of natural gas where this is separately recovered. These data are reported in 1,000 barrels/day units, and were transformed to total barrels produced per year. GDP in 1990 US dollars are from the World Resources Institute (http://earthtrends.wri.org/). All data were accessed May 15–June 15, 2011.

Here we present additional evidence that humans have approached or surpassed the capacity of the biosphere to provide essential and often non-substitutable natural resources. Figure 3 plots trends in the total and per capita use of agricultural land, fresh water, fisheries, wood, phosphate, petroleum, copper, and coal, as well as gross domestic product (GDP), from 1961 to 2008. Note that only oil, copper, coal, and perhaps fresh water show consistent increases in total consumption. Consumption of the other resources peaked in the 1980s or 1990s and has since declined. Dividing the total use of each resource by the human population gives the per capita rate of resource use, which has decreased conspicuously for all commodities except copper and coal. This means that production of these commodities has not kept pace with population growth. Consumption by the present generation is already “compromising the ability of future generations to meet their own needs.” And this does not account for continued population growth, which is projected to increase the global population to 9–10 billion by 2050 and would result in substantial further decreases in per capita consumption.

Figure 3 shows results consistent with other analyses reporting “peak” oil, fresh water, and phosphate, meaning that global stocks of these important resources have been depleted to the point that global consumption will soon decrease if it has not already done so [10],[37]. Decreased per capita consumption of essential resources might be taken as an encouraging sign of increased efficiency. But the increase in efficiency is also a response to higher prices as a result of decreasing supply and increasing demand. We have included plots for copper and coal to show that overall production of ***some*** more abundant commodities has kept pace with population growth, even though the richest stocks have already been exploited. This is typical in ecology: not all essential resources are equally limiting at any given time. Diminishing supplies of some critical resources, such as oil, phosphorus, arable land, and fresh water, jeopardize the capacity to maintain even the current human population and standard of living.

What are the consequences of these trends? Many economists and sustainability scientists suggest that there is little cause for concern, at least in the short term of years to decades. They give several reasons: i) the finite stocks have not been totally exhausted, just depleted; there are still fish in the sea, and oil, water, phosphate, copper, and coal in the ground; they are just getting harder to find and extract; ii) conservation and substitution can compensate for depletion, allowing economies to grow and provide for increases in population and standard of living; iii) production depends more on the relationship between supply and demand as reflected in price than on absolute availability; and iv) the socioeconomic status of contemporary humans depends not so much on raw materials and conventional goods as on electronic information, service industries, and the traditional economic variables of money, capital, labor, wages, prices, and debt.

There are several reasons to question this optimistic scenario. First, the fact that GDP has so far kept pace with population does not imply that resource production will do likewise. Indeed, we have shown that production of some critical resources is not keeping pace. Second, there is limited or zero scope to substitute for some resources. For most of them, all known substitutes are inferior, scarcer, and more costly. For example, there is no substitute for phosphate, which is an essential requirement of all living things and a major constituent of fertilizer. No other element has the special properties of copper, which is used extensively in electronics. Despite extensive recycling of copper, iron, aluminum, and other metals, there is increasing concern about maintaining supplies as the rich natural ores have been depleted (e.g., [38], but see [39]). Third, several of the critical resources have interacting limiting effects. For example, the roughly constant area of land in cultivation since 1990 indicates that modern agriculture has fed the increasing human population by achieving higher yields per unit area. But such increased yields have required increased inputs of oil for powering machinery, fresh water for irrigation, and phosphate for fertilizer. Similarly, increased use of finite fossil fuels has been required to synthesize nitrogen fertilizers and to maintain supplies of mineral resources, such as copper, nickel, and iron, as the richest ores have been depleted and increased energy is required to extract the remaining stocks. An optimistic scenario would suggest that increased use of coal and renewable energy sources such as solar and wind can substitute for depleted reserves of petroleum, but Figure 3 shows a similar pattern of per capita consumption for coal as for other limiting resources, and the capacity of renewables to substitute for fossil fuels is limited by thermodynamic constraints due to low energy density and economic constraints of low energy and monetary return on investment [40]–[43]. Fourth, these and similar results (e.g., [3]) are starting to illuminate the necessary interdependencies between the energetic and material currencies of ecology and the monetary currencies of economics. The relationship between decreasing supply and increasing demand is causing prices of natural resources to increase as they are depleted, and also causing prices of food to increase as fisheries are overharvested and agriculture requires increasing energy and material subsidies [2],[8],[43]. The bottom line is that the growing human population and economy are being fed by unsustainable use of finite resources of fossil fuel energy, fertilizers, and arable land and by unsustainable harvests of “renewable resources” such as fish, wood, and fresh water. Furthermore, attaining sustainability is additionally complicated by inevitable yet unpredictable changes in both human socioeconomic conditions and the extrinsic global environment [44]. Sustainability will always be a moving target and there cannot be a single long-term stable solution.

Most sustainability science focuses on efforts to improve standards of living and reduce environmental impacts at local to regional scales. These efforts will ultimately and inevitably fail unless the global system is sustainable. There is increasing evidence that modern humans have already exceeded global limits on population and socioeconomic development, because essential resources are being consumed at unsustainable rates. Attaining sustainability at the global scale will require some combination of two things: a decrease in population and/or a decrease in per capita resource consumption (see also [45]). Neither will be easy to achieve. Whether population and resource use can be reduced sufficiently and in time to avoid socioeconomic collapse and attendant human suffering is an open question.

Critics will point out that our examination of sustainability from a macroecological and natural science perspective conveys a message of “doom and gloom” and does not offer “a way forward”. It is true that humanity is faced with difficult choices, and there are no easy solutions. But the role of science is to understand how the world works, not to tell us what we want to hear. The advances of modern medicine have cured some diseases and improved health, but they have not given us immortality, because fundamental limits on human biology constrain us to a finite lifespan. Similarly, fundamental limits on the flows of energy and materials must ultimately limit the human population and level of socioeconomic development. If civilization in anything like its present form is to persist, it must take account of the finite nature of the biosphere.

## Conclusion

If sustainability science is to achieve its stated goals of “dealing with the interactions between natural and social systems” so as to “[meet] the needs of present and future generations while substantially reducing poverty and conserving the planet's life support systems”, it must take account of the ecological limits on human systems and the inherently ecological nature of the human enterprise. The human economy depends on flows of energy and materials extracted from the environment and transformed by technology to create goods and services. These flows are governed by physical conservation laws. These flows rarely balance at local or regional scales. More importantly, however, because these systems are all embedded in the global system, the flows of critical resources that currently sustain socioeconomic systems at these scales are jeopardized by unsustainable consumption at the scale of the biosphere. These ecological relationships will determine whether “sustainability” means anything more than “green”, and whether “future generations [will be able] to meet their own needs”.

## Supporting Information

Text S1 **Supplementary data.** Calculations for salmon nutrient inputs to terrestrial and riparian ecosystems.
(DOCX)

## Abbreviations

GDP: gross domestic product

## References

[1] Goodland R (1995). The concept of environmental sustainability.. Ann Rev Ecol System.

[2] Rockström J, Steffen W, Noone K, Persson A, Chapin F. S (2009). A safe operating space for humanity.. Nature.

[3] Brown J. H, Burnside W. R, Davidson A. D, DeLong J. P, Dunn W. C (2011). Energetic limits to economic growth.. BioScience.

[4] Vitousek P. M, Mooney H. A, Lubchenco J, Melillo J. M (1997). Human domination of Earth's ecosystems.. Science.

[5] Imhoff M. L, Bounoua L, Ricketts T, Loucks C, Harriss R (2004). Global patterns in human consumption of net primary production.. Nature.

[6] Haberl H, Erb K. H, Krausmann F, Gaube V, Bondeau A (2007). Quantifying and mapping the human appropriation of net primary production in earth's terrestrial ecosystems.. Proc Natl Acad Sci U S A.

[7] Pauly D, Watson R, Alder J (2005). Global trends in world fisheries: impacts on marine ecosystems and food security.. Phil Trans Roy Soc B.

[8] Worm B, Barbier E. B, Beaumont N, Duffy J. E, Folke C (2006). Impacts of biodiversity loss on ocean ecosystem services.. Science.

[9] Wackernagel M, Rees W. E (1996). Our ecological footprint: reducing human impact on the earth.. New Society Publications.

[10] Gleick P. H, Palaniappan M (2010). Peak water limits to freshwater withdrawal and use.. Proc Natl Acad Sci U S A.

[11] Brundtland G. H (1987). Our common future.. World Commission on Environment and Development.

[12] Burnside W. R, Brown J. H, Burger O, Hamilton M. J, Moses M (2012). Human macroecology: linking pattern and process in big picture human ecology.. Biol Rev.

[13] Day J. W, Boesch D. F, Clairain E. J, Kemp G. P, Laska S. B (2007). Restoration of the Mississippi Delta: lessons from hurricanes Katrina and Rita.. Science.

[14] Moses M. E, Brown J. H (2003). Allometry of human fertility and energy use.. Ecol Let.

[15] Day J. W (2009). Ecology in times of scarcity.. BioScience.

[16] Odum H. T (1971). Environment, power and society..

[17] Odum H. T (2007). Environment, power, and society for the twenty-first century: the hierarchy of energy.. Columbia University Press.

[18] Sterner R. W, Elser J. J (2002). Ecological stoichiometry: the biology of elements from molecules to the biosphere..

[19] Czúcz B, Gathman J. P, Mcpherson G. U. Y (2010). The impending peak and decline of petroleum production: an underestimated challenge for conservation of ecological integrity.. Con Biol.

[20] Hilborn R, Quinn T. P, Schindler D. E, Rogers D. E (2003). Biocomplexity and fisheries sustainability.. Proc Natl Acad Sci U S A.

[21] Hilborn R (2006). Salmon-farming impacts on wild salmon.. Proc Natl Acad Sci U S A.

[22] ADG&F (2010). Alaska historical commercial salmon catches, 1878–2010.. Alaska Department of Game and Fish, Division of Commercial Fisheries.

[23] Coupland G, Stewart K, Patton K (2010). Do you never get tired of salmon? Evidence for extreme salmon specialization at Prince Rupert harbour, British Columbia.. J Anthro Arch.

[24] Cederholm C. J, Kunze M. D, Murota T, Sibatani A (1999). A Pacific salmon carcasses: essential contributions of nutrients and energy for aquatic and terrestrial ecosystems.. Fisheries.

[25] Gende S. M, Edwards R. T, Willson M. F, Wipfli M. S (2002). Pacific salmon in aquatic and terrestrial ecosystems.. BioScience.

[26] Naiman R. J, Bilby R. E, Schindler D. E, Helfield J. M (2002). Pacific salmon, nutrients, and the dynamics of freshwater and riparian ecosystems.. Ecosystems.

[27] Schindler D. E, Scheuerell M. D, Moore J. W, Gende S. M, Francis T. B (2003). Pacific salmon and the ecology of coastal ecosystems.. Front Ecol Enviro.

[28] Schindler D. E, Leavitt P. R, Brock C. S, Johnson S. P, Quay P. D (2005). Marine-derived nutrients, commercial fisheries, and production of salmon and lake algae in Alaska.. Ecology.

[29] International Council for Science (2002). Science and technology for sustainable development.. Paris: International Council for Science.

[30] Millenium Ecosystem Assessment (2005). Ecosystems and human well-being: synthesis.. Island Press.

[31] Turner B. L, Lambin E. F, Reenberg A (2007). The emergence of land change science for global environmental change and sustainability.. Proc Natl Acad Sci U S A.

[32] Hillman T, Ramaswami A (2010). Greenhouse gas emission footprints and energy use benchmarks for eight US cities.. Environ Science & Tech.

[33] Malthus T. R (1798). An essay on the principle of population.. Prometheus.

[34] Ehrlich P. R (1968). The population bomb.. New York.

[35] Meadows D. H (1972). The limits of growth.. A report for The Club of Rome.

[36] Holdren J. P (2008). Science and technology for sustainable well-being.. Science.

[37] Nel W. P, Van Zyl G (2010). Defining limits: energy constrained economic growth.. Applied Energy.

[38] Gordon R. B, Bertram M, Graedel T. E (2006). Metal stocks and sustainability.. Proc Natl Acad Sci U S A.

[39] Kesler S. E, Wilkinson B. H (2008). Earth's copper resources estimated from tectonic diffusion of porphyry copper deposits.. Geology.

[40] Fargione J, Hill J, Tilman D, Polasky S, Hawthorne P (2008). Land clearing and the biofuel carbon debt.. Science.

[41] Hall C. A. S, Day J. W (2009). Revisiting the limits to growth after peak oil.. American Scientist.

[42] Smil V (2008). Energy in nature and society: general energetics of complex systems.. MIT Press.

[43] The Royal Society (2009). Reaping the benefits: science and the sustainable intensification of global agriculture.. London: The Royal Society.

[44] Milly P. C, Betancourt J, Falkenmark M, Hirsch R. M, Kundzewicz Z. W (2008). Stationarity is dead: whither water management?. Science.

[45] Cohen J. E (1996). How many people can the earth support?. WW Norton & Company.

[46] Foley J. A, Ramankutty N, Brauman K. A, Cassidy E. S, Gerber J. S (2011). Solutions for a cultivated planet.. Nature.

