# Supplementary material for: The Macroecology of Sustainability
Source: PLoS Biol. 2012 Jun 19;10(6):e1001345. doi: 10.1371/journal.pbio.1001345 (PMC3378595; doi:10.1371/journal.pbio.1001345)
Supplement: Text S1 — Supplementary data. Calculations for salmon nutrient inputs to terrestrial and riparian ecosystems. (DOCX) [file pbio.1001345.s001.docx]

**Supplementary data**

*Calculations for salmon nutrient inputs to terrestrial and riparian ecosystems.*

We estimated the amount of nitrogen (N), carbon (C), and phosphorous (P) that are removed from natural ecosystems and appropriated by humans due to via wild salmon harvest from Bristol Bay, Alaska. The commercial sockeye harvest in 2010 is estimated to be 83,263 tonnes. This number is based on a capture of 30.5 million individuals at 2.7 kg per fish [1]. Spawning sockeye salmon contain an average 3% fresh wet weight of N, and 0.4% fresh wet weight of P [2]. A 2.7 kg sockeye salmon contains approximately 391.8 g (14% wet weight) of C (calculated from values in [3]). On this basis, in the year 2010 the Bristol Bay fishery exported approximately 2,498 tonnes of N, 333 of P, and 11,881 of C.

*Sources of data for the city of Portland and surrounding Multnomah County*

Data on:

1) gasoline, natural gas, electricity, water, CO_2_, liquid sewage, and garbage from http://www.portlandonline.com (Accessed on 11 October 2010);

2) total domestic and international trade from http://www.aapa‑ports.org (Accessed on 12 October 2010);

3) food imports estimated based on a population of 715,000, with a metabolic rate of 120W per person consuming the equivalent of 75,161 tonnes of corn per year with energy content of 3.6kJ/g (http://www.nal.usda.gov/fnic/foodcomp/cgi-bin/list_nut_edit.pl. Accessed on 2 December 2010).

**References**

1. ADF&G (2010) Alaska Historical Commercial Salmon Catches, 1878–2010. (Alaska Department of Game and Fish, Division of Commercial Fisheries).

2. Moore, JW & Schindler, DE (2004) Nutrient export from freshwater ecosystems by anadromous sockeye salmon (*Oncorhynchus nerka*). Can J Fish Aqua Sci 61: 1582-1589.

3. Mathisen, OA et al. (1988) Recycling of marine elements transported into freshwater systems by anadromous salmon. Verh Internat Verein Limnol 23: 2249-2258.
